# Identifying Reliable and Relatable Force–Time Metrics in Athletes—Considerations for the Isometric Mid-Thigh Pull and Countermovement Jump

**DOI:** 10.3390/sports9010004

**Published:** 2020-12-31

**Authors:** Justin J. Merrigan, Jason D. Stone, W. Guy Hornsby, Joshua A. Hagen

**Affiliations:** 1 Human Performance Innovation Center, Rockefeller Neuroscience Institute, West Virginia University, Morgantown, WV 26505, USA; jason.stone1@hsc.wvu.edu (J.D.S.); william.hornsby@mail.wvu.edu (W.G.H.); joshua.hagen@hsc.wvu.edu (J.A.H.); 2 College of Physical Activity and Sport Sciences, West Virginia University, Morgantown, WV 26505, USA

**Keywords:** force plate, neuromuscular performance, strength, power, coefficient of variation, collegiate athletes

## Abstract

The purpose of this study was to evaluate intrasession reliability of countermovement jump (CMJ) and isometric mid-thigh pull (IMTP) force–time characteristics, as well as relationships between CMJ and IMTP metrics. Division I sport and club athletes (*n* = 112) completed two maximal effort CMJ and IMTP trials, in that order, on force plates. Relative and absolute reliability were assessed using intraclass correlation coefficients (ICCs) > 0.80 and coefficients of variation (CVs) < 10%. Intrasession reliability was acceptable for the majority of the CMJ force–time metrics except for concentric rate of force development (RFD), eccentric impulse and RFD, and lower limb stiffness. The IMTP’s time to peak force, instantaneous force at 150 ms, instantaneous net force, and RFD measures were not reliable. Statistically significant weak to moderate relationships (*r* = 0.20–0.46) existed between allometrically scaled CMJ and IMTP metrics, with the exception of CMJ eccentric mean power not being related with IMTP performances. A majority of CMJ and IMTP metrics met acceptable reliability standards, except RFD measures which should be used with caution. Provided CMJs and IMTPs are indicative of distinct physical fitness capabilities, it is suggested to monitor athlete performance in both tests via changes in those variables that demonstrate the greatest degree of reliability.

## 1. Introduction

An athlete’s ability to repeatedly generate large amounts of force over short amounts of time (e.g., relative to their competition) positively influences their performance in an overwhelming majority of sports [1]. Sport scientists and/or practitioners routinely assess athletes’ maximal force production (e.g., peak force) and their ability to generate force rapidly (e.g., rate of force development, RFD) as a means of assessing training adaptations and neuromuscular fatigue. Neuromuscular fatigue is commonly defined as a decrement in voluntary force generating capabilities [2]. Increased training loads and intensities, of sport training or competitions, may result in accumulated neuromuscular fatigue. These training loads and their implications must be adequately compensated for to prevent maladaptive responses to training, such as decreased power production and increased risk for musculoskeletal injury [3,4]. Given the unequivocal importance of greater force production by athletes, identifying the most effective strategies to reliably and objectively assess performance adaptations (i.e., acute and chronic changes in strength and power) is of interest in sport.

Although traditional one-repetition maximal (1-RM) testing is capable of determining maximal strength in athletes, there are concerns with its application in neuromuscular performance monitoring. Namely, 1-RMs are disruptive to the training cycles, as they are inherently fatiguing protocols [5], and as a result are counterintuitive for assessing acute or chronic neuromuscular fatigue. Further, 1-RM testing requires extensive familiarization periods to develop that specific skill set and ensure maximal capabilities are being recorded. Fortunately, due to increased availability and cost reductions, force plate testing has been adopted as a means for assessing maximal strength, as well as power output. Force plates afford sport scientists and practitioners the ability to implement a large variety of neuromuscular assessments more frequently and reliably. For example, isometric strength testing via the isometric mid-thigh pull (IMTP) is a preferable means to analyze maximal force production rather than 1-RM testing, as IMTPs are relatively simple to administer, time efficient, reduce the risk of injury, and possess high degrees of reliability under the correct testing conditions [6,7,8,9]. Additionally, despite being an isometric test (i.e., no physical movement or displacement of body segments), measures from the IMTP correlate to performance in dynamic movements of powerlifting [10,11], weightlifting [12], sprinting [13], and jumping [14,15]. Another test, the countermovement jump (CMJ), is used (often in conjunction with IMTPs) for athlete testing to identify changes in power performance [16], resiliency to fatigue [17,18,19], and risk for injury [20]. Despite the aforementioned findings of the relationship between the IMTP and dynamic assessments, the test is an assessment of strength in a static environment. Thus, the relationship between the IMTP and CMJ force–time metrics is questionable due to the distinct nature of each assessment. Therefore, more research is necessary regarding the relatedness of the two tests to improve assessment strategies for strength and conditioning practitioners.

Although testing of power output and strength on force plates may be considered the most reliable method [21,22], there are still many influential factors contributing to the reliability that must be taken into account prior to implementing testing procedures. The force plate testing software (and companion applications) typically provide a multitude of metrics from the force–time curve despite data outputs this large likely being overwhelming for most end users. The volume of metrics provided by such companies is also concerning, as some variables may be more reliable during testing and, thus, more sensitive to change in the neuromuscular status of an individual [23,24]. For example, RFD is a popular variable derived from force plate testing that previous studies have identified as an acceptable strategy for monitoring athlete performance [25,26], as well as neuromuscular fatigue [26,27]. However, other studies demonstrated that RFD was unreliable and, thus, should be used in athlete monitoring with excess caution [6,8,11]. Varying findings with respect to the IMTP force–time characteristics are a result of many factors, which include the position of the individual (i.e., knee and hip angle) [7,28], method of analysis (i.e., RFD time bands versus average RFD) [28,29], and equipment used (i.e., force plate software and hardware) [28,30]. As for the CMJ, previous literature supports the reliability of performance metrics, such as jump height and reactive strength index [31], as well as various force–time characteristics (e.g., mean and peak force and power) [32,33,34]. However, the literature on reliability assessments of CMJ variables is limited and seldom are the eccentric phase metrics assessed [19]. Since the evaluation of all of the phases of the CMJ are important when monitoring an athlete’s performance capabilities and neuromuscular fatigue, it is imperative that continued efforts are taken to highlight the reliability of these metrics and help identify which may be most beneficial for enhancing athletic performance.

To determine the consistency and agreement between metrics from two trials, the intraclass correlation coefficient is used and may be the only reliability measurement provided [11]. However, the coefficient of variation (CV) provides the typical measurement error, accounting for systematic errors that may be present in correlated data sets, unlike intraclass correlation coefficients [35]. More robust procedures would also include the confidence intervals (95% is the most common) around the reliability measures to indicate the range of reliability found during testing, since the limits above and below the average value may not be acceptable. Lastly, the literature covering the reliability of the IMTP and CMJ have typically done so separately and have included small sample sizes within a limited amount of athletic populations. This warrants further investigation on appropriate reliability testing within larger samples of underrepresented collegiate athletics. Therefore, the purpose of this study was to conduct intrasession reliability testing on the force–time characteristics of CMJ and IMTP performances of a relatively large sample of Division I (DI) Power Five National Collegiate Athletic Association (NCAA) and club sport athletes. A secondary aim of this study was to examine the relationships between the CMJ and IMTP force–time metrics. The authors hypothesized that the reliability of the force–time metrics from each test would meet acceptable reliability guidelines and there would be moderate relationships between the CMJ and IMTP.

## 2. Materials and Methods

A cross-sectional retrospective study was employed on neuromuscular performance profiling and monitoring assessments that included the IMTP and CMJ. Testing was conducted at various times throughout the year for each sport. All athletes from each sport completed the testing on the same day. Before testing, all athletes completed a short dynamic warm-up (3 sets of 20 jumping jacks, 1 set of 5 mid-thigh clean pull with 20 kg, 3 sets of 5 mid-thigh clean pull with 40 kg). Next, athletes performed 2 warm-up CMJs at 50% and 75% effort prior to completing 2 maximal effort CMJs. Then athletes performed one warm-up IMTP repetition at 50% and another at 75% effort prior to completing two maximal effort IMTP trials. The additional warm-up repetitions were used to familiarize the athletes with the testing protocols prior to data collection and allowed the researchers to gauge the athlete’s abilities to perform the movement correctly. Additional repetitions at 50% and 75% effort were allotted for those that needed more attempts to become comfortable with the movement.

### 2.1. Subjects

The study sample comprised 25 male and 87 female athletes from a collection of Division I NCAA (cheer (*n* = 18), cross country (*n* = 17), diving (*n* = 10), rowing (*n* = 34), track and field (*n* = 17)) and club (weightlifting (*n* = 16)) sports. Athletes who experienced a musculoskeletal injury in the past 6 months were excluded from the testing.

### 2.2. Countermovement Jump (CMJ)

Following the dynamic warm-up, athletes performed one CMJ at 50% effort, one CMJ at 75% effort, and two maximal effort CMJs with no arm swing. Athletes performed each jump by starting in the standing tall position with hands grasping a Polyvinyl chloride (PVC) pipe placed across their shoulders and feet placed hip width apart with one on each of the ForceDecks FD4000 Dual Force Platforms (Vald Performance, Brisbane, Queensland, Australia). The force plates were zeroed prior to the athlete stepping onto the plates, and the athletes were instructed to stand as still as possible prior to performing any jumps to determine bodyweight. The force–time curve was visually inspected to ensure limited movement occurred in the weighing phase, and a signal was provided by the software when an accurate bodyweight measure was taken. The subject was then instructed to start with equal weight distribution on both force cells. Following a 3-2-1 countdown, athletes were instructed to drop into a self-selected countermovement depth, perform a maximal effort vertical jump “as quickly and explosively as possible,” and land back onto the force plates. For each jump, verbal encouragement was provided to ensure that maximal effort was given during each attempt. Force plate data were sampled at 1000 Hz and analyzed via ForceDecks software. This software detects the initiation of movement as a 30 N deviation from the initial bodyweight calculation, eccentric to concentric phase moment as the lowest center of mass displacement, and take-off as the moment the vertical forces fall 30 N below body mass. A series of metrics from ForceDecks software’s default output were analyzed to provide the end user with reliability of a multitude of metrics of interest. These metrics are defined elsewhere [19,36,37,38] and can also be found in the ForceDecks user guide.

### 2.3. Isometric Mid-Thigh Pull (IMTP)

Following the jump testing, athletes performed a series of IMTPs on a different set of the same dual force plates via a testing rack specifically designed to fix the bar at any desired height above the custom-designed power rack (Sorinex, Inc., Irmo, SC, USA). Similar to process used for the CMJ, the force plates were zeroed prior to the athlete stepping onto the plates, and bodyweight was initially determined prior to the athletes performing one IMTP at 50% effort, one IMTP at 75% effort, and two IMTPs at maximal effort. For IMTP testing, the apparatus was set to place the athlete in a fixed, standard, power-pulling position, as identified in previously published work (130–140° and 145° for the knee and hip angles, respectively) [7,36]. The bar height was set to half the thigh length (50% of the distance between the greater trochanter and lateral epicondyle of the knee), and a goniometer was used to confirm that knee and hip angles were within acceptable ranges, according to prior work, while each subject stood on the force platform [6,7]. The bar was completely immoveable to ensure accurate readings by reducing the “slack” in the bar that would result in signal noise. After positioning, athletes’ hands were strapped to the bar using wrist straps and standard athletic tape to remove any effect of grip strength on IMTP. All repetitions were separated by one minute of rest. Athletes were instructed to pull upward on the bar “as quickly and explosively as possible,” and maintain maximal effort for three seconds. Force plate data were again sampled at 1000 Hz and analyzed via ForceDecks software. The metrics assessed for the IMTP were selected based on the same conditions as the CMJ and are described elsewhere [28,36].

### 2.4. Statistical Analysis

An acceptable relative reliability threshold based on intraclass correlation coefficients (ICC_2,k_) was >0.80 [39]. Acceptable absolute reliability was considered as coefficients of variation (CVs) <10%, which were calculated as percentages of the standard deviation divided by the mean of trials [39]. Additionally, the standard error of the measurement was calculated as an indicator of reliability using the *sem()* function in the *‘rel’* package. To evaluate the relationships between IMTP and CMJ force–time metrics, Pearson’s correlation coefficients were computed using the following effect size determinants; weak, *r* = 0.10–0.40; moderate, *r* = 0.41–0.70; strong, *r* > 0.71 [40]. All statistical procedures were conducted using R, version 3.6.2 (R Core Team, Vienna, Austria; https://www.R-project.org), except the CVs, which were calculated in Excel (Microsoft Inc., Redmond, WA, USA).

## 3. Results

Intrasession reliability was deemed acceptable for many of the countermovement jump force–time metrics. However, concentric RFD and lower limb stiffness did not meet acceptable reliability thresholds (CVs > 10%; ICCs < 0.80; Table 1 and Table 2). Additionally, eccentric braking impulse, eccentric braking RFD, and eccentric deceleration RFD were all outside of acceptable CV ranges, whereas eccentric duration had high absolute (CV = 6.41%) but low relative reliability (ICC = 0.76; Table 1 and Table 2). For the IMTP, variables of most concern included time to peak force and instantaneous force at 150 ms, as well as all instantaneous net force and RFD measures, according to failure to meet either or both reliability thresholds (Table 3 and Table 4). Statistically significant weak to moderate relationships existed between commonly scaled CMJ and IMTP force–time metrics, with the exception of CMJ eccentric mean power not being related with IMTP performances (Table 5).

## 4. Discussion

The primary findings of this investigation were (1) the identification of CMJ and IMTP force–time metrics, via ForceDecks, that fell below the acceptable reliability thresholds (ICC > 0.80, %CV > 10%) and (2) the low to moderate relationships between CMJ and IMTP force–time metrics. These findings, from a range of NCAA DI and club sport athletes, add to the limited research on the reliability of athlete monitoring via CMJ and IMTP assessments beyond the most common metrics. Further, the low to moderate relations between the two tests may indicate distinct differences in the physical fitness capabilities each test measures (i.e., maximal strength versus explosive power) and highlights the usefulness of including both in human performance monitoring protocols.

The current findings of acceptable intrasession absolute and relative reliability for commonly assessed CMJ force–time metrics are in line with previous literature examining the no arm swing CMJ [19,31,32,33,34]. Due to the variety of techniques, such as tape on the wall or jump contact mats [41], jump height is often regarded as the most practical and commonly used metric from CMJ testing. There are many ways to calculate jump height that typically include strategies that incorporate flight time or the impulse–momentum theorem [21,37]. In agreement with prior findings from NCAA basketball athletes [19], all current methods for calculating jump height were deemed reliable and, as a result, may be used to detect change. However, jump height calculations from flight time had slightly improved relative reliability compared to impulse–momentum and impulse–displacement theorems. These methods of calculating jump height are well defined in prior literature [21,42,43], with the main difference between the integration techniques (i.e., impulse–momentum and impulse–displacement) being the integrated metric (i.e., velocity or displacement). Many others have also found high levels of reliability for jump height estimations from flight time, which may also be more practical, since jump mats can be used in place of force plates, if necessary [31,34,41,44]. Further, although the gold standard is the impulse–momentum theorem, incorrect identifications of the take-off moment by 2–3 milliseconds can lead to 2% variations in velocity and displacement calculations [45]. These miscalculations would lead to approximately 0.9 cm absolute errors in jump height calculations [46]. Thus, although the impulse–momentum theorem may be considered as the gold standard for jump height derivations, the use of certain equipment in practical settings may result in slightly lower reliability, thus making estimations from flight time, to some extent, more favorable.

Other concentric performances during the CMJ achieved acceptable relative (ICC) and absolute (CV) reliability, with the exception of RFD. Indeed, previous literature reported that RFD metrics were unreliable, with CVs ranging from 16% to 54% [19,31,32]. However, similar to RFD, the rate of power development (RPD) may prove a useful alternative during CMJ assessments, but warrants additional investigation [19,32,33,47]. The RPD during the CMJs concentric phase was considered reliable in the current study and is in agreement with the limited prior findings (ICC = 0.89–0.99; CV = 4.6–9.9%) [19,32,33,47]. Although RFD is often regarded as a key performance indicator for athletic performances [25,26,27], multiple studies have now called into question its inherent reliability. This makes RFD less than adequate for monitoring neuromuscular readiness and fatigue, as large changes will be necessary to overcome the standard error in the measurement (i.e., the changes observed in RFD may or may not be indicative of changes in neuromuscular status). However, it is important to note that average measure ICCs and sampling frequencies above 1000 Hz may improve the error of less reliable variables, although these scenarios may become less practical [31,33]. Therefore, RPD may be a preferred metric over RFD during CMJ testing. Other metrics that may be of interest during the concentric phase of the CMJ are modified reactive strength index (RSI-mod), mean and peak forces and power, impulse, velocity, and duration, all of which were reliable in the current study. Although some of these variables are seldom investigated, prior findings also indicate reliability for peak and mean velocity, force, and power, as well as duration and impulse measures [19,31,32,33]. Lastly, a common metric for evaluating an athlete’s explosiveness and ability to create substantial forces in a short period of time, RSI-mod has been considered reliable and thus a useful monitoring tool [19,48,49].

Despite the hypothesized associations with neuromuscular fatigue [32], the eccentric phase of the countermovement jump has limited evidence in regard to the reliability of its metrics [19]. However, in agreement with the limited research [19,50], the eccentric phase’s mean braking and deceleration force, overall mean and peak force and power, dip-depth and peak velocity were all considered to have acceptable intrasession absolute and relative reliability. Yet, eccentric baking impulse, braking and deceleration RFD, eccentric duration, and lower limb stiffness were all considered unreliable, in agreement with prior research [19]. Of note, the eccentric RFD variables met acceptable relative reliability, but failed to meet absolute reliability thresholds, which was previously found as well [31]. Others have also demonstrated that the eccentric metrics may be more reliable under no arm swing CMJ protocols [19]. More research is encouraged to understand the reliability of the eccentric metrics, as well as their sensitivity to detecting neuromuscular fatigue. These efforts will enable end-users to fully grasp their relevance for understanding neuromuscular function and monitoring performance adaptations (or lack thereof) longitudinally.

For the IMTP, absolute impulse, instantaneous forces, and absolute and net peak forces were considered reliable in the current study. Similar to the CMJ, peak and instantaneous forces and impulse measures were previously found to be reliable [11]. However, RFD measures during IMTPs are often considered unreliable metrics [11,28,29]. If RFD is utilized, it is encouraged to use time epochs (i.e., 0.−200 milliseconds) [28,29]. Yet, prior research indicated that there was acceptable relative reliability during longer epochs (i.e., 200 milliseconds) compared to shorter epochs (i.e., 90 milliseconds), but unacceptable absolute reliability at all epochs [11]. Likewise, in the current study, time epochs above 100 milliseconds elicited acceptable relative reliability, but no time epochs met the absolute reliability thresholds. To improve the reliability of IMTP metrics, such as RFD, it is suggested that practitioners carefully and consistently abide by the recommended guidelines for conducting the testing, particularly ensuring the bodyweight calculation is accurate and there is no countermovement prior to initiating the pull [28,36]. Either scenario would result in misidentification of the initiation of the pull and drastically alter the RFD calculations. However, even with careful monitoring and implementation of these testing protocols in the current study, RFD failed to meet acceptable reliability. Thus, the calculations used to identify the initiation of the pull may also be influential to the reliability of RFD, while not impeding peak force calculations, and must be considered across software manufacturers. Interestingly, the instantaneous net forces were not reliable, in disagreement with instantaneous absolute forces, which highlights the need to explore reliability functions of all measures prior to their use. Thus, from a reliability standpoint, caution should be taken when monitoring instantaneous net force and RFD during IMTPs. Although absolute impulse, instantaneous, and peak force suggest utility, more research is warranted to explore their relation to sport performance and neuromuscular fatigue.

From the current findings, CMJ and IMTP metrics are significantly correlated, but the strength of these relationships was low to moderate (i.e., *r* < 0.50). Although strength may drive explosive capabilities, strength and explosive strength are independent qualities [11], which may suggest why these low relationships existed. The IMTP is a measure of maximal isometric strength capabilities, while the CMJ is an explosive dynamic movement. Thus, the importance of strength for power production capabilities, but distinctness between the CMJ and IMTP, cumulatively contributes to the low to moderate correlations among the tests’ metrics. For example, in astronauts, no relationships were found between IMTP and squat jumps [11], although strong correlations have existed between IMTP variables and jumping performances in more traditional sport athletes [51,52]. Statistically significant correlations of similar magnitudes were found in a variety of NCAA DI sport athletes when comparing allometrically scaled IMTP metrics to unweighted countermovement jump heights [51]. Yet, in recreationally trained males, and adolescent basketball athletes, the relationship between IMTP peak force and jump height were strong (*r* = 0.72 to 0.94) [52,53], while IMTP RFD was not related with jump performances [53]. Thus, for less trained individuals, the relationship between the IMTP and CMJ may be greater, but other factors such as sample sizes or metrics used may also explain the divergent findings. Others have demonstrated that while IMTP force characteristics may not be related with jump height, absolute peak force and impulse of the IMTP were related to CMJ peak force and power [54]. Lastly, despite some finding weak relationships of IMTP RFD with jump performances [51,53], IMTP RFD may be useful for monitoring neuromuscular fatigue, as previous research suggested it was more sensitive than peak forces [26,27], although longer epochs (100–200 milliseconds) are recommended [28,29]. Overall, in the current athletes, the IMTP and CMJ likely test different domains of physical fitness capabilities and should both be included in performance monitoring programs.

It is important to note the limitations of the current study, as the athletes in the current study were relatively weaker and less skilled in the current movements compared to competitive weightlifters in prior literature [26] and were performing these assessments for their first time. Although speculative, the generally homogenous force data may contribute to the weak to moderate relationships between CMJ and IMTP performances. Heterogeneous data sets with greater disparity between relatively weaker and stronger individuals are more likely to yield stronger correlations, as the stronger individuals are more likely to jump higher and vice versa, which would falsely inflate the strength of the correlation between the IMTP and CMJ. Moreover, these findings suggest that within power-based sports at this level of play (Power 5 athletes, excluding the club sport athletes) there may be groups of athletes lacking a solid foundation of strength. These findings present important training ramifications, as relatively weaker athletes will more likely benefit from improvements in strength prior to training power [55]. Further, certain variables within the IMTP (e.g., RFD) may have improved reliability when the participants have more familiarity with the movement [22]. Thus, it is important for coaches to monitor reliability and continuously coach the correct positions (both in the weight room and for IMTP testing). The poor reliability found in RFD (and other time-sensitive force data) should not deter coaches from embarking on IMTP testing, as maximal strength is a necessary assessment tool for longitudinal monitoring in strength-power athletes [56]. Further, although a current limitation may be the minimum familiarity each athlete had performing these tests, it is important to understand which metrics from each test will be reliable under conditions where extensive familiarization periods are unlikely.

## 5. Conclusions

There is an abundance of force–time metrics for CMJ and IMTP assessments, which vary to a large degree in terms of their reliability. The findings presented herein suggest that RFD measures may be unreliable in both the CMJ and IMTP for athletes of similar training statuses. However, RPD and absolute impulse (with time bands) were reliable and therefore useful metrics to use in place of RFD during the CMJ and IMTP, respectively. Lastly, the CMJ and IMTP metrics likely test different physical fitness capabilities in trained individuals, as shown by the weak relationships between the two tests. It should be noted that substantially more research is necessary to understand how to best utilize these tests and further assist in identifying the best metrics from each test, which is dictated by their reliability and sensitivity to change. However, it is encouraged that both the CMJ and IMTP be used in performance monitoring programs, since each tool reliably assesses different physical performance constructs (i.e., maximal strength versus power). For example, IMTP peak and instantaneous force, but not RFD, was reliable in this study and thus useful as measures of maximal strength when athletes are testing for the first time. Yet, for initial assessments of explosiveness, reliable metrics from the CMJ can be used.

## Figures and Tables

**Table 1:** Countermovement jump eccentric force–time curve metrics and their reliability.

	Trial 1 Mean ± SD	Trial 2 Mean ± SD	%CV (LCI, UCI)	ICC (LCI, UCI)	SEM (LCI, UCI)
Dip Depth (cm)	−30.57 ± 8.48	−31.23 ± 9.82	7.66	0.90	2.91
			(5.00, 10.32)	(0.87, 0.93)	(2.50, 3.33)
ECC Braking Impulse (Ns)	60.84 ± 72.54	60.11 ± 67.76	**14.53**	0.98	8.92
			**(11.36, 17.70)**	(0.98, 0.99)	(7.72, 10.11)
ECC Braking RFD (N∙s^−1^)	4780 ± 2060	4924 ± 2446	**10.86**	0.87	815.1
			**(9.26, 12.45)**	(0.83, 0.90)	(697.2, 933.0)
ECC Decel. Impulse (Ns)	95.56 ± 50.31	95.26 ± 51.46	6.34	0.98	7.65
			(4.13, 8.55)	(0.97, 0.98)	(6.54, 8.75)
ECC Decel. RFD (N∙s^−1^)	5661 ± 2890	5841 ± 3256	**10.38**	0.87	815.1
			**(8.56, 12.20)**	(0.83, 0.90)	(697.2, 933.0)
ECC Duration (ms)	479.3 ± 76.0	479.2 ± 93.7	6.41	**0.76**	**41.61**
			(4.50, 8.31)	**(0.60, 0.82)**	(35.77, 47.45)
ECC Mean Braking Force (N)	843.8 ± 193.2	846.6 ± 199.2	3.38	0.96	40.97
			(2.71, 4.05)	(0.94, 0.97)	(35.33, 46.61)
ECC Mean Decel. Force (N)	1222 ± 309	1224 ± 329	3.43	0.96	64.14
			(2.71, 4.15)	(0.95, 0.97)	(55.08, 73.20)
ECC Mean Force (N)	689.9 ± 149.7	689.8 ± 149.6	0.06	0.99	0.648
			(0.05, 0.08)	(0.99, 0.99)	(0.557, 0.739)
ECC Mean Power (W)	438.0 ± 142.1	442.5 ± 151.1	6.83	0.93	39.98
			(4.74, 8.92)	(0.90, 0.95)	(34.30, 45.66)
ECC Peak Force (N)	1573 ± 406	1584 ± 429	3.34	0.97	78.17
			(2.70, 3.97)	(0.95, 0.97)	(67.49, 88.86)
ECC Peak Power (W)	1223 ± 525	1239 ± 602	9.24	0.98	121.1
			(6.74, 11.74)	(0.98, 0.99)	(102.7, 139.5)
ECC Peak Velocity (m∙s^−1^)	−1.23 ± 0.30	−1.23 ± 0.33	6.26	0.87	0.112
			(4.04, 8.47)	(0.83, 0.91)	(0.095, 0.129)

Bold values indicate that the metric failed to meet acceptable reliability based on coefficient of variation (CV) of >10% and intraclass correlation coefficient (ICC) of <0.70. SD: standard deviation; UCI: upper confidence interval; LCI: lower confidence interval; SEM: standard error of measurement; RFD: rate of force development; decel.: deceleration.

**Table 2:** Reliability of countermovement jump concentric force–time and performance metrics.

	Trial 1 Mean ± SD	Trial 2 Mean ± SD	%CV (LCI, UCI)	ICC (LCI, UCI)	SEM (LCI, UCI)
CON Duration (ms)	2.80 ± 0.50	2.86 ± 0.58	4.67	0.88	19.50
			(3.84, 5.49)	(0.84, 0.91)	(16.80, 22.19)
CON Impulse (Ns)	158.46 ± 45.23	158.77 ± 45.82	1.62	0.98	5.94
			(1.03, 2.20)	(0.98, 0.99)	(4.93, 6.94)
CON Mean Force (N)	1273 ± 319	1265 ± 321	2.15	0.99	36.30
			(1.81, 2.48)	(0.98, 0.99)	(31.83, 40.76)
CON Mean Power (W)	1633 ± 544	1630.7 ± 553	3.20	0.98	77.78
			(2.55, 3.86)	(0.97, 0.99)	(66.85, 88.71)
CON Peak Force (N)	1605 ± 407	1612 ± 418	2.91	0.98	63.84
			(2.44, 3.37)	(0.97, 0.98)	(55.77, 71.92)
CON Peak Velocity (m∙s^−1^)	2.38 ± 0.26	2.38 ± 0.28	1.44	0.89	0.091
			(0.89, 1.99)	(0.85, 0.92)	(0.075, 0.106)
CON RFD (N∙s^−1^)	720 ± 1138	759 ± 1244	**76.45**	**0.57**	783.4
			**(66.86, 86.03)**	**(0.45, 0.66)**	(662.6, 904.3)
CON RPD (W∙s^−1^)	14,673 ± 6521	14,308 ± 6599	7.15	0.95	1552
			(6.08, 8.21)	(0.93, 0.96)	(1342, 1762)
Jump Height (Flight) (cm)	27.30 ± 6.41	27.46 ± 6.36	2.92	0.97	1.03
			(2.49, 3.35)	(0.96, 0.98)	(0.905, 1.15)
Jump Height (Imp-Dis) (cm)	26.01 ± 6.40	26.17 ± 7.09	3.21	0.87	2.44
			(2.09, 4.33)	(0.82, 0.90)	(2.01, 2.87)
Jump Height (Imp-Mom) (cm)	25.96 ± 6.39	26.13 ± 7.09	3.20	0.87	2.44
			(2.08, 4.33)	(0.83, 0.90)	(2.01, 2.87)
Lower Limb Stiffness (N∙m^−1^)	4971 ± 3081	6181 ± 10,643	**10.21**	**0.35**	6345
			**(7.59, 12.82)**	**(0.21, 0.48)**	(5176, 7515)
Peak Power (W)	2979 ± 972	2955 ± 982	2.48	0.98	121.1
			(1.88, 3.08)	(0.98, 0.99)	(102.7, 139.5)
RSI-modified (m∙s^−1^)	0.37 ± 0.11	0.37 ± 0.12	5.81	0.91	0.036
			(4.87, 6.75)	(0.87, 0.93)	(0.031, 0.041)

Bold values indicate that the metric failed to meet acceptable reliability based on coefficient of variation (CV) of >10% and intraclass correlation coefficient (ICC) of <0.70. SD: standard deviation; UCI: upper confidence interval; LCI: lower confidence interval; SEM: standard error of measurement; RFD: rate of force development; decel.: deceleration.

**Table 3:** Isometric mid-thigh pull force–time curve metrics and their relative and absolute reliability.

	Trial 1 Mean ± SD	Trial 2 Mean ± SD	%CV (LCI, UCI)	ICC (LCI, UCI)	SEM (LCI, UCI)
Absolute Impulse 50 ms (Ns)	46.0 ± 16.7	45.1 ± 12.6	7.0	**0.67**	8.55
			(5.1, 9.0)	**(0.57, 0.74)**	(7.09, 10.01)
Absolute Impulse 100 ms (Ns)	101.2 ± 38.4	100.4 ± 31.7	7.4	**0.76**	17.31
			(5.6, 9.2)	**(0.68, 0.82)**	(14.44, 20.17)
Absolute Impulse 150 ms (Ns)	170.4 ± 67.9	170.1 ± 59.7	8.2	0.83	26.677
			(6.6, 9.9)	(0.77, 0.87)	(22.53, 30.82)
Absolute Impulse 200 ms (Ns)	253.5 ± 102.9	253.8 ± 92.8	8.5	0.87	34.98
			(6.0, 10.0)	(0.83, 0.91)	(29.99, 40.06)
Force at 50 ms (N)	983.9 ± 374.4	972.2 ± 297.1	7.1	**0.72**	178.1
			(5.3, 9.0)	**(0.64, 0.79)**	(148.0, 208.2)
Force at 100 ms (N)	1243.0 ± 539.9	1251.3 ± 502.3	9.9	0.84	210.1
			(8.2, 11.7)	(0.78, 0.88)	(179.5, 240.7)
Force at 150 ms (N)	1524.3 ± 678.9	1536.2 ± 640.5	**10.2**	0.90	207.3
			**(8.6, 11.8)**	(0.87, 0.93)	(181.0, 233.6)
Force at 200 ms (N)	1793.6 ± 775.0	1807.3 ± 714.7	9.5	0.92	211.2
			(8.0, 11.0)	(0.89, 0.94)	(185.9, 236.4)
Net Force at 50 ms (N)	99.5 ± 100.3	105.7 ± 103.7	**28.4**	**0.75**	51.34
			**(24.7, 32.1)**	**(0.67, 0.81)**	(43.54, 59.13)
Net Force at 100 ms (N)	358.7 ± 314.0	384.8 ± 348.9	**31.4**	0.82	140.9
			**(27.0, 35.8)**	(0.76, 0.87)	(122.6, 159.1)
Net Force at 150 ms (N)	640.0 ± 480.1	669.7 ± 486.4	**26.7**	0.86	181.0
			**(22.7, 30.8)**	(0.81, 090)	(159.4, 202.6)
Net Force at 200 ms (N)	909.3 ± 591.6	940.8 ± 554.9	**22.2**	0.87	206.4
			**(18.5, 25.9)**	(0.83, 0.90	(181.6, 231.2)
Net Peak Vertical Force (N)	1905.9 ± 805.1	1949.8 ± 818.1	7.1	0.94	192.1
			(5.7, 8.5)	(0.93, 0.96)	(164.5, 219.9)
Peak Vertical Force (N)	2790.2 ± 984.6	2816.3 ± 983.7	3.5	0.99	116.1
			(3.0, 4.1)	(0.98, 0.99)	(102.6, 129.7)
Start Time to Peak Force (s)	2.61 ± 1.30	2.57 ± 1.41	**31.2**	**0.48**	0.97
			**(26.8, 35.6)**	**(0.36, 0.60)**	(0.86, 1.09)

Bold values indicate that the metric failed to meet acceptable reliability based on coefficient of variation (CV) of >10.0% and intraclass correlation coefficient (ICC) of <0.80. SD: standard deviation; UCI: upper confidence interval; LCI: lower confidence interval; SEM: standard error of measurement; RFD: rate of force development.

**Table 4:** Isometric mid-thigh pull force–time curve metrics and their relative and absolute reliability.

	Trial 1 Mean ± SD	Trial 2 Mean ± SD	%CV (LCI, UCI)	ICC (LCI, UCI)	SEM (LCI, UCI)
RFD 30 ms (N∙s^−1^)	1307.5 ± 2020.9	1229.9 ± 986.5	**26.6**	**0.41**	1216.1
			**(23.0, 30.3)**	**(0.28, 0.54)**	(1000.7, 1431.5)
RFD 50 ms (N∙s^−1^)	1990.2 ± 2006.1	2113.7 ± 2075.0	**28.4**	**0.75**	1026.85
			**(24.6, 32.1)**	**(0.67, 0.81)**	(870.86, 1182.8)
RFD 50–100 ms (N∙s^−1^)	5182.3 ± 4577.9	5582.7 ± 4995.1	**35.4**	0.81	2121.8
			**(30.3, 40.5)**	(0.74, 0.85)	(1856.1, 2387.5)
RFD 100 ms (N∙s^−1^)	3586.3 ± 3140.1	3848.2 ± 3489.3	**31.4**	0.82	1408.6
			**(27.0, 35.9)**	(0.76, 0.87)	(1226.1, 1591.1)
RFD 100–150 ms (N∙s^−1^)	5626.6 ± 3969.9	5696.2 ± 3485.0	**28.9**	**0.73**	1934.1
			**(23.8, 34.0)**	**(0.65, 0.80)**	(1670.0, 2198.3)
RFD 150 ms (N∙s^−1^)	4266.4 ± 3200.7	4461.1 ± 3227.6	**26.8**	0.86	1203.9
			**(22.7, 30.8)**	(0.81, 0.90)	(1060.1, 1347.7)
RFD 150–200 ms (N∙s^−1^)	5385.0 ± 2888.7	5466.0 ± 2790.8	**28.1**	**0.64**	1706.6
			**(23.5, 32.7)**	**(0.70, 0.84)**	(1496.6, 1916.5)
RFD 200 ms (N∙s^−1^)	4546.1 ± 2957.8	4703.8 ± 2774.3	**22.2**	0.87	1031.70
			**(18.5, 25.9)**	(0.83, 0.90)	(908.08, 1155.3)

Bold values indicate that the metric failed to meet acceptable reliability based on coefficient of variation (CV) of >10.0% and intraclass correlation coefficient (ICC) of <0.80. SD: standard deviation; UCI: upper confidence interval; LCI: lower confidence interval; SEM: standard error of measurement; RFD: rate of force development.

**Table 5:** Relationships between scaled isometric mid-thigh pulls and countermovement jumps.

	Force at 50 ms (N/kg)	Force at 100 ms (N/kg)	Force at 150 ms (N/kg)	Force at 200 ms (N/kg)	Peak Force (N/kg)
Concentric Mean Power (W/kg)	0.26 *	0.42 *	0.44 *	0.44 *	0.43 *
Concentric Peak Force (N/kg)	0.22 *	0.35 *	0.37 *	0.38 *	0.35 *
Eccentric Mean Power (W/kg)	0.08	0.16	0.15	0.15	0.18
Eccentric Peak Force (N/kg)	0.20 *	0.34 *	0.35 *	0.36 *	0.36 *
Jump Height (Imp-Mom) (cm)	0.23 *	0.39 *	0.40 *	0.41 *	0.46 *
RSI-modified (m/s)	0.27 *	0.41 *	0.43 *	0.42 *	0.35 *

Bold values indicate the relationship is of moderate strength, while * indicates statistical significance. Correlation coefficients are classified as weak, r = 0.10–0.40; moderate, r = 0.41–0.70; strong, r > 0.71.

## Data Availability

Data sharing is not applicable to this article.
